# GMOs or non-GMOs? The CRISPR Conundrum

**DOI:** 10.3389/fpls.2023.1232938

**Published:** 2023-10-09

**Authors:** Aftab Ahmad, Amer Jamil, Nayla Munawar

**Affiliations:** ^1^ Center for Advanced Studies in Agriculture and Food Security (CASAFS), University of Agriculture Faisalabad, Faisalabad, Pakistan; ^2^ Department of Biochemistry, University of Agriculture, Faisalabad, Pakistan; ^3^ Department of Chemistry, College of Science, United Arab Emirates University, Al-Ain, United Arab Emirates

**Keywords:** CRISPR-Cas, gene editing, GMOs, GM regulations, transgenic plants

## Abstract

CRISPR-Cas9, the “genetic scissors”, is being presaged as a revolutionary technology, having tremendous potential to create designer crops by introducing precise and targeted modifications in the genome to achieve global food security in the face of climate change and increasing population. Traditional genetic engineering relies on random and unpredictable insertion of isolated genes or foreign DNA elements into the plant genome. However, CRISPR-Cas based gene editing does not necessarily involve inserting a foreign DNA element into the plant genome from different species but introducing new traits by precisely altering the existing genes. CRISPR edited crops are touching markets, however, the world community is divided over whether these crops should be considered genetically modified (GM) or non-GM. Classification of CRISPR edited crops, especially transgene free crops as traditional GM crops, will significantly affect their future and public acceptance in some regions. Therefore, the future of the CRISPR edited crops is depending upon their regulation as GM or non-GMs, and their public perception. Here we briefly discuss how CRISPR edited crops are different from traditional genetically modified crops. In addition, we discuss different CRISPR reagents and their delivery tools to produce transgene-free CRISPR edited crops. Moreover, we also summarize the regulatory classification of CRISPR modifications and how different countries are regulating CRISPR edited crops. We summarize that the controversy of CRISPR-edited plants as GM or non-GM will continue until a universal, transparent, and scalable regulatory framework for CRISPR-edited plants will be introduced worldwide, with increased public awareness by involving all stakeholders.

## 1 Introduction

**Figure 1:**
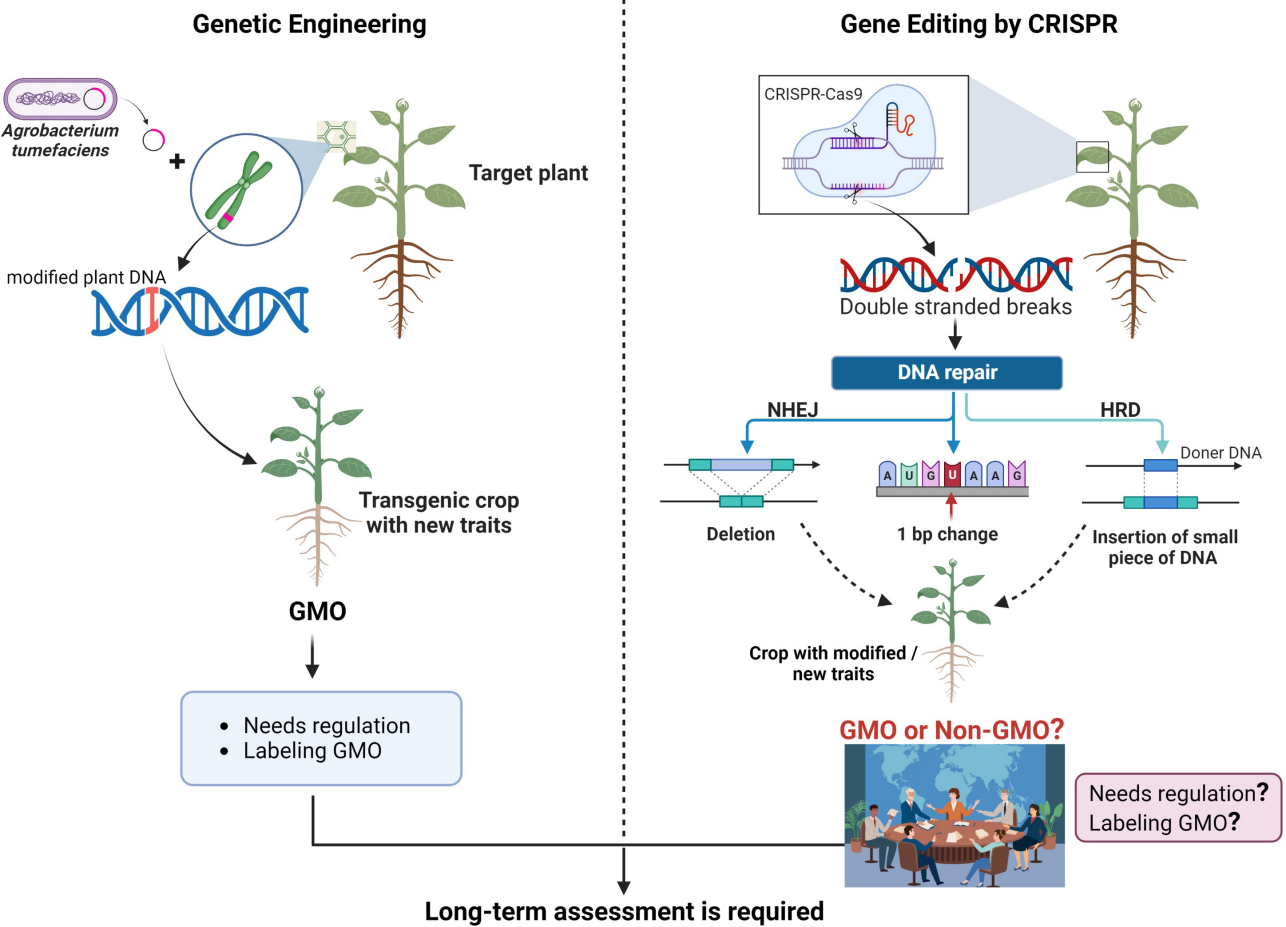
Comparison of traditional genetic engineering and CRISPR-Cas editing technique. Genetic engineering is used to introduce new traits in crops through the insertion of a gene from different species and produces GMOs which need strict regulations and product labeling (left side). CRISPR-Cas9 introduces a DSB in the target DNA and let the cell repairs itself to introduce small changes such as indels, which can be used to improve gene function(s). A small piece of DNA could also be introduced from the same species, or an existing gene could be modified without inserting any foreign DNA in the plant genome, to introduce a new trait in plants using CRISPR-Cas9 (right side).

Extreme weather patterns and climate variability have a negative impact on global food security for the growing world population. We must find new solutions and discover new technologies to meet the promises of food and nutritional security at the global level. CRISPR-Cas9 (Clustered Regularly Interspaced Short Palindromic Repeats/Cas associated protein 9), a gene editing tool, has been predicted as a revolutionary discovery of the 21st century to reshape the genomic landscape of not only bacteria, but also animals and plants to achieve our goals in food security, therapeutics, and human health. Therefore, this technique grabbed the attention of scientists and private companies to engineer agricultural crops with climate resilience, disease resistance, and better nutritional profile. Similarly, CRISPR-Cas technology has been adopted universally for translational applications in human health, therapeutics, and product development. CRISPR-Cas as a gene editing tool uses endonuclease (known as Cas) recruited by a 20 bp guide RNA (gRNA) to introduce double-stranded breaks (DSBs) at a precise target sequence (complementary to gRNA), which results in specific and targeted genetic modification during DNA repair mechanisms (**Figure 1**; Hou et al., 2023). CRISPR has become a gold standard to create novel genetic variations by installing precise DNA modifications to introduce new and improved traits in animals and plants (Zaidi et al., 2019).

Applications of the CRISPR-Cas system in model and crop plants have become a routine to address emerging problems of insect/pests, disease resistance, and heat and drought tolerance in plants (He and Zhao, 2020). CRISPR-Cas has enabled precise changes in the genome, in a way never possible with traditional transgenic approaches. The technique is overcoming barriers and has the potential to fulfill the early promises of genetic engineering such as plants with higher yield, better biotic and abiotic resistance, disease and pest resistance, less water requirement, and more nutritious food (Bailey-Serres et al., 2019). CRISPR-Cas based techniques have been successfully used for proof-of-concept studies in model and crop plants for better yield and quality, herbicide resistance, and environment sustainability. Compared to traditional genetic engineering, where genetic modifications in the host genome were always random, CRISPR-Cas based modifications are precise, predictable, inheritable, and sometimes without introducing any external gene sequence in the host-genome. However, critics argue that both CRISPR technology and classic genetic engineering use the same transformation method (Agrobacterium and biolistic) to introduce genetic modifications and marker genes for the selection of transformants, which has created a debate among the scientific community if CRISPR edited crops should be considered genetically modified (GM) or not. However, marker genes could be removed from the CRISPR edited crops by crossing or transgene killer technology (Yubing et al., 2019). Similarly, off targets are also one of the potential concerns associated with CRISPR, especially in therapeutics and human health applications.

While CRISPR holds an incredible potential to rewrite the genomic landscape of agricultural crops, how CRISPR edited crops will be regulated will determine the future of this revolutionary technology. Regulation of CRISPR edited crops has lagged the pace of development and regulatory authorities are facing challenges in keeping with complexities and risk evaluation. The pace of regulation is slower than the rate of scientific advancement in many jurisdictions, leading to the perception of gridlock in the system. The world community is divided over the policies, legal status, and regulatory requirements of the CRISPR edited crops. For example, the US and EU (European Union) have different opinions on the regulatory status of these crops. Nonetheless, the United States Department of Agriculture (USDA) decided to deregulate CRISPR edited crops, especially SDN1 (Site-Directed Nuclease 1) and SDN2 modified crops, because they do not contain any foreign DNA (transgene) and the modifications did not involve any pesticidal properties. USDA determined that CRISPR editing is equivalent to conventional breeding in some instances, thus does not require strict GMO (Genetically Modified Organism) regulations. For example, CRISPR-edited mushrooms developed by Yinong Yang at Pennsylvania State University in 2015 were approved by-passing the strict regulations of GMOs (Waltz, 2016a). As SDN1 and SDN2 modified plants are indistinguishable from conventionally bred plants, so SDN1 and SDN2 modifications are considered as non-GM plants by different countries such as the US, Argentina, and Brazil. On the other hand, European Union (EU) regulations follow a more precautionary approach and consider all plants, modified through either gene editing or genetic engineering, as GM even if they are free of any transgene. This decision may have serious implications on research, development, and commercialization of CRISPR technology in Europe (Hjort et al., 2021). Compared with SDN1 and SDN2 crops, SDN3 crops are always considered as GM and must pass through strict GMO regulations and risk assessment in many countries of the world. So far, the regulation of CRISPR edited crops varies significantly among different countries and this mosaicism in the regulation is partly due to the different definitions of genetic modifications and genetically modified organisms (GMOs) by various regulatory authorities and the world community (Wolt et al., 2016a). The lack of adequate regulatory and legal guidance on CRISPR edited crops has led to a debate on classification and legal status, particularly concerning whether these crops should be considered as GM or non-GM. In addition, the distinction between SDN1 and SDN2 modifications and point mutations created through spontaneous mutations or conventional methods is also an important part of the debate. Whether SDN1 and SDN2 modification and point mutations should be considered different from conventional GM crops or from each other, is an important discussion in the scientific community, regulatory authorities, and different stakeholders. It is also worth noting that none of the countries have designed a *de-novo* regulation of CRISPR edited crops, but the current regulations for CRISPR edited crops are based on the already existing regulatory framework of GMOs in different countries (Turnbull et al., 2021). Therefore, it is important to explore the current definition of genetically modified (GM) and gene edited (GE) plants to carefully determine the difference between GM and non-GM crops. A conducive, universal, and transparent regulatory framework, along with better social and consumer acceptance of CRISPR edited crops could have positive impacts on food security, environmental sustainability, and faster crop development.

## 2 Genetically modified or non-genetically modified?

Genetic modification is a broader term that involves different methods, such as traditional breeding and modern gene editing methods, to modify genetic composition of plants or animals to achieve the desired traits. Genetic engineering is a specific type of genetic modification that involves deliberate manipulation of an organism’s genome through biotechnology techniques such as genetic engineering and gene editing. Transgenic plants are always produced through genetic engineering approaches by artificially inserting an exogenous DNA stretch into a plant genome, usually from an unrelated species to achieve a desired trait. For example, *Bt* gene(s) from *Bacillus thuringiensis* was transferred in different crops such as cotton and corn by genetic engineering, to improve these crops against insect attacks (Sarker et al., 2019; **Figure 1**). These plants, which undergo artificial DNA modifications to confer desired traits are called as genetically modified (GM) plants. Generally, scientists and regulatory authorities consider a plant as GM, if there has been a transfer of gene(s) in its genome, from distantly related species such as bacteria, insects, or animals, using biotechnology techniques (Rani and Usha, 2013). Conventional breeding and genetic engineering involve random and uncontrolled mutagenesis for introducing genetic changes to achieve desired traits in crops.

GM organisms have been used for various applications in basic and applied research. However, genetically modified crops and their respective food products have negative perceptions among the scientific community and the public due to their potential health concerns and lateral gene flow to non-target organisms, which could raise unknown environmental issues (Funk and Kennedy, 2016). In addition, the use of antibiotic resistant marker genes for the selection of transformants was also a concern in their public acceptance. Since the first release of GM crops in 1994, more than 70 countries have adopted GM crops for cultivating or importing to date (Srivastava et al., 2011; Borém et al., 2014; Wunderlich and Gatto, 2015; Turnbull et al., 2021; ISAAA Inc, ). During 2020, 94% of soybean crops in the USA were GM having herbicide tolerance, while other major GM crops cultivated in the USA were cotton and corn (Edwards, 2020; Kumar et al., 2020; Brookes, 2022).

Despite the success of GM crops in improving agricultural productivity and addressing several other challenges, developers and agriculture companies are struggling for their better public acceptance and global commercialization because of their possible impact on the environment and human health (Raman, 2017; Ahmad et al., 2021). Majority of the transgenic crops contain genes from unrelated species, transferred through Agrobacterium, to improve crops against insects or to withstand herbicides. These crops could induce pest resistance by releasing toxins in soil and destroying crop biodiversity, thus could have an adverse environmental impact. GM crops having bacterial or insect genes have raised health concerns such as allergic reactions which have been reported in humans in different countries (Zhang et al., 2016). It is important to note that while there have been reports of allergic reactions and health concerns, there is lack of scientific evidences linking these concerns directly to consumption of GM crops (Dunn et al., 2017). Scientific studies have not supported these claims about GM crops and regulatory agencies such as FDA (Food and Drug Administration), EFSA (European Food Safety Authority) and WHO (World Health Organization) conduct extensive regulatory and safety assessments of GM crops before their approval for commercial use. Therefore, GM crops approved by these regulatory agencies for commercial use are considered safe for human consumption.

GM crops containing DNA from other species must undergo a lengthy regulation and approval process (**Figure 1**; Halford, 2019). Therefore, multinational companies are exploring alternative biotechnological methods to improve crops without involving transgene transformation from unrelated species. For example, topical application of dsRNA has been used as a potential insecticidal approach for insect control in crops (Lu et al., 2023). Meanwhile, during the past decade, modern gene editing techniques involving various nucleases such as zinc finger nucleases (ZFNs), transcription activator-like effector nucleases (TALENs), and CRISPR-Cas9 using engineered nucleases emerged as new tools for site-specific DNA modifications in the genome (Li et al., 2020a). Although, ZFNs and TALENs have been used for targeted gene editing in plants, the CRISPR-Cas9 has emerged as a more powerful and versatile tool due to its simplicity, efficiency, and modularity. The detailed mechanism of action, applications, and limitations of different genome-editing techniques have been reported elsewhere (Munawar and Ahmad, 2022).

It is noteworthy that among different gene editing tools, CRISPR-Cas9 gained rapid popularity in crop improvement programs because of its simple design, less time consumption, cost-effectiveness, good reproducibility, high efficiency, precise targeting, and diverse applications. As shown in **Figure 1**, CRISPR-Cas9 simply introduces DSBs at the target site in the genome. DSBs in the genome provoke natural DNA repair systems such as non-homologous end joining (NHEJ), or homology-directed repair (HRD) in the cell, consequently introducing indels or precise insertions, respectively, at the target site in the host genome (Lin et al., 2014). While, CRISPR-Cas9 has been extensively used in plant gene editing, with remarkable efficiency and precision, many other CRISPR-Cas systems are also available, each with its unique properties and applications. These alternate CRISPR-Cas systems offer different advantages and capabilities that can be used for specific purposes and applications. For example, CRISPR-Cas12a recognizes a T-rich PAM and produces staggered ends at the DSB site. While Cas9 and Cas12 have been used specifically for DNA editing, CRISPR-Cas13 is an RNA editing system which can modulate expression at RNA level, without introducing any permanent change in the genome. Similarly, CRISPR-Cas14 offers a unique advantage in targeting ssDNA (single stranded DNA) instead of the usual dsDNA targeted by Cas9 and Cas12. CasX and CasY are relatively new members of the CRISPR-Cas family, which are being characterized for their potential applications in gene editing. CRISPR mediated base editing (BE) systems allow precise change from one nucleotide (A, T, G, C) into another, converting one DNA base pair into another, without causing DSB in the DNA. For example, CRISPR based adenine base editor (ABE) and cytosine base editor (CBE) can result in the conversion of adenine (A) to inosine (I) and cytosine (C) to uracil (U) respectively. The cellular repair system then converts inosine to guanine (G) and uracil to thymine (T), thus resulting in targeted and precise changes to the individual DNA bases in the genome. CRISPR based BE systems have shown a great promise to install precise modifications in the genome of crops to develop new crop varieties with improved traits (Gaudelli et al., 2017; Molla and Yang, 2019). CRISPR mediated prime editing (PE) is another innovative technique that allows precise insertion, deletion, or substitutions at the target site in the genome without causing DSBs. PE offers a greater control over genetic modifications, allowing researchers to make specific changes by directly writing new DNA sequences in the genome. It is a rapidly advancing field of research and its applications are being explored in crops for introducing new and desired traits (Anzalone et al., 2020; Li et al., 2020b). Very recently, CRISPR-like systems, such as OMEGA and Fanzor, have been identified in eukaryotes which may further improve gene editing with reduced off targets and improved efficiency (Altae-Tran et al., 2021; Saito et al., 2023). Desired traits in crops can be achieved through CRISPR-Cas by utilizing nature generated genetic variations present in the genomes of non-modified plants. For example, SDN1 and SDN2 genome edited plants which can be generated through targeted modifications of the plant’s own genes without permanently integrating DNA in the plant genome may arise from spontaneous mutations or can be achieved through classical breeding. So, CRISPR edited SDN1 and SDN2 plants are generally characterized as non-GM, because they are not based on introducing new genes in the host plant to obtain desirable traits, thus making them more acceptable as compared to the plants generated through conventional genetic engineering (Abdallah et al., 2015; Jones et al., 2022). Compared to GM crops, non-GM crops have certain benefits associated with those such as faster development, precise modifications in the genome, absence of transgenes, predictable outcomes, and reduced regulatory challenges. In contrast, SDN3 crops are produced by providing a donor template containing large DNA fragment such as transgene or cis-gene and are regulated under strict GMO regulations (Georges and Ray, 2017).

Regardless of the rapid development of CRISPR-Cas technology and its potential applications in editing the genomes of model and crop plants since 2013 (Upadhyay et al., 2013), only a few CRISPR edited crops have reached the market so far (Hazman, 2022). Although CRISPR-Cas has been presented as a precise genome editing technique, off-targets remain a potential concern in the scientific community, especially in therapeutics, human health, and product development (Cribbs and Perera, 2017; Omodamilola and Ibrahim, 2018). However, off target effects can be mitigated by several approaches ranging from careful gRNA design to modifications in experimental strategies and different molecular diagnostic tools to detect and quantify off targets. In plants, off targets are not a major concern, because any off-target mutations are likely to be segregated out during subsequent breeding and selection steps (Yee, 2016). Several researchers have generated CRISPR edited plants without any off targets (Nekrasov et al., 2017). Scientists have also expressed concerns about CRISPR based gene drives due to their potential environmental impact as these will be difficult to control once released into the environment (Mueller, 2019). However, CRISPR/Cas based gene editing is continuously evolving with new tools, having diverse functions which could be helpful in addressing these challenges in the future (Zong et al., 2022). Nevertheless, the main challenges to bring CRISPR edited crops in the market are consumer acceptance, a universal regulatory system, transparent policies, and public awareness about gene edited plants.

Both CRISPR mediated gene editing and traditional genetic engineering lead to genetic modifications, however, CRISPR based modifications are very precise, predictable, free of transgene, and sometimes as small as a single base pair (bp) editing in the entire genome. In addition, unlike transgenic methods where foreign genetic elements are always present such as marker genes (Zhang et al., 2020), CRISPR-Cas editing does not necessarily introduce foreign DNA elements in the host genome, but it depends upon the type of CRISPR-Cas reagents (Cas9 and sgRNA) and their delivery methods. The use of Cas9/sgRNA plasmid DNA for CRISPR-Cas gene editing is an efficient and simple method, nevertheless, it is not free of limitations (Liu et al., 2017; Eoh and Gu, 2019). The large size of plasmids (9–19 kb) and their permanent integration in the host genome may result in continuous expression, leading to higher off-target effects. Using *in-vitro* transcribed mRNA offers several advantages such as transient expression, reduced off-targets, and less risk of integration in the genome (Li et al., 2014; Jiang et al., 2017; Glass et al., 2018; Son and Park, 2022). But the poor stability of mRNA and reduced efficiency of gene editing with mRNA are the major limitations of this approach. Ribonucleoprotein (RNP) based CRISPR-Cas system has also been used for CRISPR applications in plants, which results in obtaining transgene-free plants, which are not considered GM according to one concept (Távora et al., 2022). For example, Cas9 protein and sgRNA as mRNA were delivered through lipofection in plants to obtain transgene free CRISPR edited plants (Zhang S. et al., 2021). The use of RNP does not introduce any foreign DNA sequence permanently into the plant genome and reduces off-target effects (Liu et al., 2017; Eoh and Gu, 2019). However, as the biotechnology process is used to install new modifications in the genome of the host plant, and the genome is “modified” it may be considered GM according to another concept (Kim and Kim, 2016).

Depending on the repair outcomes of DSBs, CRISPR mediated modifications are classified into three main categories: site-directed nuclease types 1, 2, and 3, known as SDN1, SDN2, and SDN3, respectively (Wolt et al., 2016b). In SDN1, DSBs are repaired through NHEJ repair system which introduces indels (adds or deletes nucleotides) without using any repair template. In SDN2, a microhomologies-mediated repair template is used to add, delete, or replace very few (2-10) specific nucleotides at the target site (Xue and Greene, 2021). The resultant plants in both SDN1 and SDN2 are indistinguishable from conventionally bred plants, and thus could be considered non-GM. Therefore, most countries like the US, Japan, India, Australia, and Ecuador consider SDN1 and SDN2 modified plants safe and do not regulate them under conventional GM regulations (Tachikawa and Matsuo, 2023). In SDN3, a repair template through homologous recombination is used to insert a gene segment or whole gene at the targeted site, resulting in transgenic or cis-genic plants, consequently, triggering regulatory oversight depending on the nature and origin of the introduced DNA segment (Friedrichs et al., 2019). CRISPR modifications are classified as SDN1, SDN2, and SDN3 based on the repair mechanism of DSB, and different countries have developed their regulatory framework to distinguish between these modifications. The legal and regulatory status of these crops vary from country to country, depending upon the definition of GMOs, existing regulations for GMOs, and the specific techniques used. For example, the US, Argentina, and Japan consider SDN1 and SDN2 edited plants as non-GM and deregulate them, however SDN3 edited plants are considered on case-by-case basis. In contrast, EU, and New Zealand, which use process-based triggers for regulating GM crops, determine CRISPR edited crops are same as conventional genetically modified plants, having transgenes. Therefore, every country has a distinct regulatory framework for CRISPR modified crops to address GM and GE controversies (Turnbull et al., 2021). In the following section, we have discussed the regulatory oversight of CRISPR edited crops in different countries.

## 3 Current global regulations of CRISPR edited crops

The world community has a strong division over regulatory triggers for GM crops (Sprink et al., 2016). There are two main regulatory triggers for the regulation of GM crops in the world: a product-based system and a process-based, while some countries are following a mixture of these two approaches, tailored to their needs (Sprink et al., 2016). The USA follows product-based regulations for GM crops, whereas the EU regulatory system is based on the method by which a product is made, without considering the traits expressed in the product (Jones, 2015; Wolt et al., 2016b; Jones et al., 2022). Compared with a process-based trigger, the product-based trigger is considered more straightforward, aligned with WTO, and reliable because any risk posed by the modified plant will be arising from the product itself but not from the method or technique used to generate it (Dederer and Hamburger, 2019). While Canadian regulation for genetically modified crops is based on plants with novel traits (PNTs). A novel trait in plants could be introduced through conventional breeding, genetic engineering, or gene editing.

CRISPR crops are touching global markets and some of those, especially SDN1 and SDN2 crops, have been approved by the US, Argentina, Japan, and Brazil (means that these crops are no longer considered regulated under the Plant Protection Act and can be marketed without the same level of regulatory oversight as traditional GM crops) bypassing the strict regulatory framework of conventional GMOs (Stoye, 2016; Unglesbee, 2016; Grossman, 2019; Menz et al., 2020). Deregulation of SDN1 and SDN2 crops in several countries in the world may accelerate the development of new crops with improved traits. However, SDN3 crops are still considered as GMOs and regulated under the conventional GM framework. Although CRISPR edited crops, especially SDN1 and SDN2, could be free of any transgene or foreign genetic elements (promoter or terminator), the debate persists on how to regulate those crops and what precautionary measures are required before these crops appear in the market. Nonetheless, the difference in the scientific and legal communities on regulatory triggers of GM crops is also hindering the legislation for gene edited crops in other countries like Australia and European countries that insist to regulate GE crops like GMOs (Hamburger, 2019).

GMOs pass through strict regulations in many countries of the world, especially in the European countries due to the presence of foreign gene(s) and their potential risks to human health (Zhang et al., 2016). Public trust could be built only by providing clear and reliable scientific and legal information about the CRISPR-Cas technique and its possible impact in comparison to transgenic GM crops (Kato-Nitta et al., 2019). The current regulations for GM and GE crops and the responsible agencies for these regulations in different countries are shown in **Table 1**.

**Table 1:** Genome editing regulations in different countries and their regulatory agencies.

Country	Regulatory Agency	Genome Editing Regulations	SDN1	SDN2	SDN3	Approved Crops	References
United States	USDA, APHIS, FDA, EPA	New SECURE Rules (2020) Coordinated Framework for the Regulation of Biotechnology; Plant Protection Act; National Environmental Policy Act; Federal Insecticide, Fungicide, and Rodenticide Act (FIFRA	Deregulated	Deregulated	Case-by-case different	Maize, Tomato, Soybean, Mushroom, Flax	Wolt and Wolf, 2018; Ahmad et al., 2021; Turnbull et al., 2021
Argentina	Argentine Biosafety Commission (CONABIA)	Resolution No. 173/15 (2015)	Deregulated	Deregulated	Deregulated (if not transgenic)	–	Lema, 2019; Whelan et al., 2020
Australia	Food Standards Australia New Zealand (FSANZ)	Gene Technology Amendment (Measures No. 1) to regulations (2019)	Deregulated	Regulated	Regulated	–	Turnbull et al., 2021
New Zealand	Environmental Protection Authority (EPA), Food Standards Australia New Zealand (FSANZ)	Hazardous Substances and New Organisms (HSNO) Act 1996	Regulated	Regulated	Regulated	–	Turnbull et al., 2021
Japan	The Ministry of Agriculture, Forestry and Fisheries (MAFF), the Ministry of Health, Labour and Welfare (MHLW), the Ministry of Environment (MOE)	Handling Procedures MHLW: Food Hygiene Handling. Procedures for Food and Additives Derived from Genome Editing (2019); Notification by MOE: Handling of organisms obtained through the use of genome editing technology that do not fall under “genetically modified organisms” as defined in the Cartagena Act(2019)	Deregulated	Deregulated	Regulated	Tomato	Igarashi and Hatta, 2018; Menz et al., 2020
Brazil	National Technical Commission for Biosafety (CTNBio)	Normative Resolution No. 16 (2018)	Deregulated	Deregulated	Deregulated (if not transgenic)	–	Gatica-Arias, 2020
Canada	Canadian Food Inspection Agency (CFIA)	Food and Drug Regulations (Division 28 of Part B) Directive 94-08 (CEPA) Seeds Act; Part V of the Seeds Regulations Directive 95-03, Guidelines for the Assessment of Novel Feeds: Plant Sources Health. Canada’s Guidelines for the Safety Assessment of Novel Foods Volume II	Case-by-case (by novelty)	Case-by-case (by novelty)	Case-by-case (by novelty)	–	McHughen, 2016; Smyth, 2017
EU		Directive 18/2001/EC (2001) after court decision in case C-528/16	Regulated	Regulated	Regulated	–	Menz et al., 2020
Israel	The National Committee for Transgenic Plants	Seed regulations 5765– 2005 (Genetically Modified Plants and Organisms) (2005) after decision of the National Committee for Transgenic plants (2017)	Deregulated	Deregulated	Transgenic: Regulated Cisgenic: Deregulated	–	Turnbull et al., 2021
Colombia	Colombian Agricultural Institute (ICA)	Resolution No. 00029299 (2019)	Case-by-case	Case-by-case	Deregulated (if not transgenic)	–	Turnbull et al., 2021
Honduras		Agreement SENASA 008-2019 (2019)	Case-by-case	Case-by-case	Deregulated (if not transgenic)	–	Gatica-Arias, 2020
Chile	Ministry of Agriculture’s Agricultural and Livestock Services (SAG)	Introduction of methodological procedure (2017)	Deregulated	Deregulated	Deregulated (if not transgenic)	–	Turnbull et al., 2021
China	Ministry of Agriculture and Rural Affairs (MARA), National Biosafety Committee (NBC),	Regulations on Administration of Agricultural Genetically Modified Organisms Safety	Under development	Under development	Under development	Soyabean	Cao, 2018; Chen and Dai, 2020
India	Indian Ministry of Science and Technology (2020), Genetic Engineering Appraisal Committee (GEAC)	Draft Document on Genome Edited Organisms: Regulatory Framework and Guidelines for Risk Assessment (2020)	Under development	Under development	Under development	–	Turnbull et al., 2021
Pakistan	National biosafety committee		Under development	Under development	Under development	–	Babar et al., 2020

In the USA, USDA regulates GM and CRISPR edited crops if those contain a foreign DNA sequence from other species (Entine et al., 2021). As described earlier, the USA follows the product-based regulation of GMOs without any concern about the method used, focusing only on the traits expressed (McHughen, 2016; Sprink et al., 2016; Smyth, 2017). USDA statement about the regulation of GMOs “Under its biotechnology regulations, USDA does not currently regulate, or have any plans to regulate plants that could otherwise have been developed through traditional breeding techniques as long as they are developed without the use of a plant pest as the donor or vector and they are not themselves plant pests” (Grossman, 2019). Under this definition, base editing, deletion, and insertion from related species would not be regulated as GM by USDA. Therefore, several CRISPR-edited crops have been deregulated and approved for commercialization, bypassing the existing strict regulations of GMOs (Grossman, 2019). USDA in 2018 declared that it “does not currently regulate or have any plans to regulate” CRISPR-edited crops (Duensing et al., 2018). Although USDA is deregulating CRISPR-edited plants, experts still suggest that they need to consider regulatory, governance, and ethical oversight of CRISPR edited crops (Cotter and Perls, 2018). In addition, CRISPR based gene drives, multiplex gene editing crops, and crops with permanently integrated markers and Cas9 gene should pass through strict regulations (Arora and Narula, 2017). Similarly, in Israel, National Committee for Transgenic Plants decided not to regulate GE crops, however, product developers must demonstrate that no foreign DNA has been inserted in the plant genome (Menz et al., 2020). Canadian regulatory system is based on plants with novel traits (PNTs) and remained unchanged with the emergence of gene edited crops (Smyth, 2017). PNTs is a flexible and product-oriented system in which plant products are subjected to regulation depending upon the novelty of the trait, irrespective of their production method. However, under PNTs regulation, all products are evaluated for their allergenicity and toxicity (Eckerstorfer et al., 2019). Argentina follows a flexible regulatory system based on the presence or absence of a transgene. If a transgene or a new combination of genetic material is present, it will be considered GMO, while if no transgene is used, the product will be considered non-GMO. Similarly, if a transgene was used but has been removed from the final product through crossing, the product would be considered non-GMO (Entine et al., 2021). In contrast to the USA, where genetic material determines the status of a plant as GMO, the EU defines GMO as any organism created through genetic modification technology (McHughen, 2016). In addition, labeling of products as GM foods is mandatory in the EU and any food must also be labeled as GMO if the source ingredients are attained from GMO, even if no GMO is present in the final product (Castellari et al., 2018). Based upon these regulations, the European Court of Justice (ECJ) ruled out that CRISPR edited plants would be considered GMOs and would pass through existing process-based regulations for GMOs (Hamburger, 2019). Similarly, the Australian government has decided to regulate the gene edited crops with foreign DNA integrated into the genome as GMO (especially constructed by SDN2 and SDN3), however, gene edited products with no foreign DNA present in them (constructed by SDN1) could be considered safe and exempted from regulations (Zhang Y. et al., 2021). So far, China has no formal regulation for gene edited crops, but the country has invested heavily in gene editing technology, showing its intention to develop its own gene edited crops (Hundleby and Harwood, 2022). While Chinese authorities are monitoring carefully how the USA is regulating gene edited crops, it could be expected that China would have flexible regulations for gene edited crops. So, none of the countries have developed an entirely new regulatory framework for gene edited crops, but most of these frameworks are based on existing GMO regulations. Therefore, with the current lack of adequate legal guidance and a universal scalable regulatory system for CRISPR edited crops, the situation will remain uncertain about the regulatory status of gene edited crops (Pixley et al., 2022). Although the deregulation of CRISPR edited crops and self-determination of exemption status (SECURE rule) by USDA has accelerated trait improvement through this cutting-edge technology both in the public and private sectors, opponents are also concerned that companies may mislead regulatory authorities and market their GM crops through this exemption from regulatory oversight. In addition, unintentional modifications may pass unnoticed by this self-determining exemption, consequently posing risks. Moreover, self-determination of exemption by the GM crops developers would have a significant impact on consumers, especially regarding the safety of food products.

In our opinion, the rapid rise in CRISPR-Cas technology and its ability to redesign the genomic landscape for crop improvement needs a clear, universal, and scalable regulatory framework to accommodate future developments in CRISPR such as synthetic biology applications, multiplex gene edited crops and gene drives in crops. In addition, the regulatory and legal status of point mutations and base edited CRISPR crops (free of any transgene), must be defined to establish a clear and consistent regulatory framework for CRISPR edited crops. In addition, it will help researchers, developers and farmers to understand their requirements for commercialization and consumer acceptance of their crops. Point mutations in the crop genome that improve food quality could be exempted from strict GM regulations. For example, the deletion of a few base pairs from polyphenol oxidase (PPO) gene reduces 30% activity of the enzyme resulting in brown-resistant mushrooms (Waltz, 2016a). Similarly, waxy corn having high amylopectin was produced by knocking out *Wx1* gene (Waltz, 2016b). *Camelina sativa* (false flax) was also modified with improved omega-3 oil content (Waltz, 2018). All these studies highlight the potential of CRISPR-Cas technology to produce transgene free crops with small modifications in their genome to improve the existing traits as well as introducing new traits in the crops to meet the challenges of food security. The production of nicotine-free non-transgenic tobacco using CRISPR-Cas9 could be utilized to facilitate people in their efforts to reduce their nicotine addiction (Schachtsiek and Stehle, 2019). It is worth mentioning here that a strong link between specialized scientists, the public, and legislation authorities is required to assist policymakers in developing unambiguous and transparent regulations for GE crops and make edited crops reliable and acceptable for consumers. Nonetheless, the long-term effects of GM and GE crops should be evaluated before bringing these crops to the commercial market.

## 4 Conclusion

Although CRISPR-Cas technology holds an incredible potential for developing new crops with improved traits, however, several challenges persist, such as efficient delivery of CRISPR reagents, consumer acceptance, intellectual property rights, trait stacking and combinatorial editing, and different jurisdictions of CRISPR edited plants, to fully realize the potential of this revolutionary technology. Moreover, the difficulty in detection and traceability of CRISPR edited SDN1 and SDN2 crops is an important consideration in regulation, labelling, and commercialization of these crops. With the rapid rise in CRISPR technology, the old paradigms and regulatory frameworks of conventional GMOs should be reevaluated to accommodate new developments such as transgene free CRISPR edited crops with precise and point mutations. Thus, it is important to enhance international coordination among all stakeholders including scientists, policy makers, regulatory authorities, politicians, farmers, industry representative, and public to revisit the regulatory framework. All stakeholders must be engaged for globally harmonized definitions and regulatory policies for precise genetic modifications and increased public awareness to address the unique challenges about regulation of gene edited crops. It will be worth observing the European Commission’s anticipated new policies in the coming years. EU policies about GE crops will have a high impact on R&D and innovation of technology. Development of a universal, transparent, scalable, and mutually agreed-upon regulation for gene edited plants, holds a tremendous potential to address the global challenges related to food security, sustainable agriculture, and the growing world population. At the same time, ethical responsibilities like self-determination of exemption of CRISPR modified crops by farmers and agricultural companies should be controlled by strict monitoring. The long-term effect of GE crops should not be ignored for a healthier and sustainable environment.

## Author contributions

NM and AJ conceived the idea. AA wrote the manuscript. NM finalized the manuscript. All authors contributed to the article and approved the submitted version.
